# New York Letter

**Published:** 1891-11

**Authors:** 


### Correspondence.

### NEW YORK LETTER.

NEW YORK, October 29th, 1891.

A very interesting case of puerperal eclampsia was brought to the notice of the staff of the New York Polyclinic Hospital a few days ago. The patient was a married woman 30 years of age, the mother of four children, all of whom are living and in good health. She was seven months pregnant, when a few days ago she was suddenly seized with convulsions and was taken for treatment to the Polyclinic Hospital. Measures were at once taken for her delivery and she was placed under the influence of chloroform to that end. The physicians were considerably alarmed when they observed her respiration suddenly ceased and artificial respiration had to be resorted to. This was supplemented by hypodermic injections of brandy and one-hundredth of a grain of nitro-glycerine. Immediately after the delivery of the fœtus, her temperature rose to 103 4-10 and her pulse was feeble and about 140, continuing in that condition the greater part of the night. During the night she was given 4 drachms of brandy hypodermically, with one one-hundredth of a grain of nitro-glycerine every three hours, and twenty-five grains of chloral by the rectum twice during the night. Constant catheterization had to be resorted to. Her urine showed from the date of her admittance about ninety per cent. of albumen and hyaline casts. The day following her delivery, the amount of albumen was about 50 per cent, gradually diminishing until at the present time it is about twenty per cent. She remained unconscious from the time of her admission into the hospital until the following morning, a period of some eighteen hours in all. She is at present writing convalescing and shows every indication of steady improvement.

Dr. Wyeth recently performed craniotomy on a boy 14 years of age at the New York Polyclinic,for the cure of epileptiform convulsions. The patient had been the subject of epileptic seizures from childhood. There was an indirect history of traumatism, but this was not believed to have any bearing on his present trouble. The operation consisted in the removal of a section of bone three and a half inches long and three-quarters of an inch in width, from the left side of the skull. The patient has gotten along very nicely since the operation, and has not developed any one of his former symptoms. Sufficient time has not, however, elapsed to form a possible opinion on the permanent effects of the operative procedure in this boy's case.

Dr. H. Marion Sims recently spoke as follows on the subject of hemorrhoids:

"Of all the methods of treatment employed for the cure of hemorrhoids, the simplest, easiest and most efficacious I have found to be the old method of ligation. Within the past few years there has been brought to the notice of the profession a plan of treating this condition by means of carbolic acid injection. I must say, after noting the effects of this plan of treatment, that one can never be certain what effect will be produced within a hemorrhoidal tumor by the injection of carbolic acid. Used in the most careful manner in which it is possible to use it, the acid is always liable to produce sloughing. I remember the case of a young man who had been treated by an expert in the use of carbolic acid injections and who sent for me one night. I went to his house and found him in great agony. On examination of the rectum I found three inches of the gut had sloughed away to such an extent that it took three weeks subsequent treatment before healing was effected."

Dr. Gerster, of this city, is still hopeful of the ultimate good effects of the Koch lymph treatment of tuberculosis. While in Berlin last spring he saw some cases of lupus with terrible deformity that had been treated by the lymph, and had been so influenced by its use that large, ulcerating surfaces that had remained open for years, notwithstanding other modes of treatment, had cicatrized. Sections of this granulating surface were made and were found to contain bacilli under the microscope. Of course, such a condition as this cannot be considered a cure, but, Dr. Gerster asks, looking at it from the standpoint of the general practitioner and not of the pathologist, is not the patient left in a much better condition physically than he was before, even though this cure were to continue but for a period of six months? He considers tuberculine a wonderful remedy, and thinks that the time may yet arrive when it might be converved into a genuine blessing to mankind.

Dr. A. L. Loomis, speaking at a recent clinical lecture on paroxysmal cardiac dyspnœa, said there is nothing that so certainly induces degenerative changes in the cardiac muscles and consequent dilatation of the cavities of the heart, which are the cause of this condition, as the intemperate use of alcohol. It is evident therefore, that to treat this condition the excessive use of alcohol must be stopped, but never suddenly or entirely, for a moderate amount of alcohol is essential to the nutritive processes in a chronic alcoholic subject: The diet should be in every instance restricted to the use of milk, meat and a small amount of bread. Sugars and starches should be avoided, and the quantity of food limited. Flannel should be worn next to the skin and the surface of the body should never be allowed to become chilled. Next to diet the most important thing is exercise in the open air; commencing in a moderate way, it should be daily increased until the individual is able to take long walks without fatigue, avoiding elevations and going upstairs. The medical treatment resolves itself into alkalies, the different preparations of iron, in combination with small doses of digitalis. In alcoholic subjects strychnine should be combined with the iron. All of these drugs should be given in small doses and continued for a long time. The first thing in the management of the paroxysm is to give the patient plenty of fresh air; second, to keep him in a semi-recumbent posture, and third, to apply artificial heat to the surface of the body. The only two medicinal agents that have any positive control over a paroxysm are the nitrite of amyl and nitro-glycerine. After one paroxysm has occurred, nitro-glycerine should be given whenever the premonitory symptoms of an attack are present. During a paroxysm nitrite of amyl carefully administered will give at least temporary relief. J. P. R.

